# The Importance of the Monitoring of Resuscitation with Blood Transfusion for Uterine Inversion in Obstetrical Hemorrhage

**DOI:** 10.1155/2015/269156

**Published:** 2015-09-30

**Authors:** Seishi Furukawa, Hiroshi Sameshima

**Affiliations:** Department of Obstetrics & Gynecology, Faculty of Medicine, University of Miyazaki, Miyazaki 889-1692, Japan

## Abstract

*Objective*. The aim of this study was to describe critical care for obstetrical hemorrhage, especially in cases of uterine inversion. *Study Design*. We extracted data for six patients diagnosed with uterine inversion concerning resuscitation. *Results*. The shock index on admission of the six patients was 1.6 or more on admission. Four of the six experienced delay in diagnosis and received inadequate fluid replacement. Five of the six experienced delay in transfer. Five of the six underwent simultaneous blood transfusion on admission, and the remaining patient experienced a delay of 30 minutes. All six patients successfully underwent uterine replacement soon after admission. One maternal death occurred due to inappropriate practices that included delay in diagnosis, delay in transfer, inadequate fluid replacement, and delayed transfusion. Two patients experiencing inappropriate practices involving delay in diagnosis, delay in transfer, and inadequate fluid replacement survived. *Conclusion*. If a delay in diagnosis occurs simultaneously with a delay in transfer and inadequate fluid replacement, failure in providing a prompt blood transfusion may be critical and result in maternal death. The monitoring of resuscitation with blood transfusion for uterine inversion is essential for the improvement of obstetrical care.

## 1. Introduction

An autopsy registry survey in Japan indicated that half of the maternal deaths are due to obstetric hemorrhage [1]. In our local survey, uterine inversion accounts for 3.3% of all pregnant women who received any blood transfusion for obstetrical hemorrhage, and the cases of uterine inversion included one maternal death. The incidence of uterine inversion was relatively low, but the prognosis was very poor [2].

Early recognition of uterine inversion is indispensable for early replacement of the inversion to stop the bleeding, and the assessment of vital signs and preparation to prevent hemorrhagic shock are also required. As a consequence, several management protocols for uterine inversion have been proposed [3, 4]. However, despite widespread familiarity with management practices and attention regarding hemorrhage, maternal death still occurs in cases of uterine inversion [2, 5]. The review of individual cases of obstetrical care for uterine inversion is definitely needed to improve maternal outcomes.

Many reports have outlined risk factors concerning the development of uterine inversion [6–8] or useful measures for the replacement of an inverted uterus [5]. However, there is still a lack of clarity regarding the effect of resuscitation failure on mortality and morbidity concerning uterine inversion. In this study, we reviewed the medical records of pregnant patients with uterine inversion. We extracted data concerning resuscitation including condition of the women on admission (shock index: SI), diagnosis, transfer to perinatal center, and clinical practices including uterine replacement, fluid replacement, and blood transfusion therapy. We then compared differences concerning the resuscitation practice within the group of cases involving uterine inversion.

## 2. Materials and Methods

This study was undertaken retrospectively and obtained approval (#2013-135) from a suitably constituted ethics committee at our institution. We retrospectively reviewed the medical charts of pregnant women with uterine inversion that were admitted to the Perinatal Center of the University of Miyazaki, the Miyazaki Medical Association Hospital, the Fujimoto General Hospital, or the Nichinan Prefectural Hospital from January 2007 to December 2013. In this study, there were cases of uterine inversion that were also included in our previous study [2]. The Perinatal Center of the University of Miyazaki is a tertiary center, whereas the other hospitals are secondary perinatal centers. These centers mainly deal with high-risk pregnancy. Most parturitions involving low-risk cases are overseen by private clinics. In cases involving an obstetrical emergency, women are referred to a high-order perinatal center dependent on the level of severity [9]. Access to blood products except for platelets is ensured within 60 minutes after a request by any of the centers. A small stock of blood products is available at secondary perinatal centers.

We reviewed the medical records of pregnant patients with uterine inversion and the following maternal characteristics were collected: age, parity (primipara), history of abortion, gestational age at birth, birth weight (g), use of vacuum extraction, and attempted manual removal of the placenta. We checked the following factors related to resuscitation for uterine inversion in each case: inborn or outborn delivery (i.e., private clinic), presence of immediate diagnosis of uterine inversion, SI on admission, presence of prompt transfer to the perinatal center after diagnosis, presence of proper practices including immediate uterine replacement on admission to the perinatal center, adequate fluid replacement before admission to the perinatal center, prompt blood transfusion after admission to the perinatal center, estimated total blood loss within the first 24 hours following delivery, and presence of ICU care. SI was defined as heart rate/systolic blood pressure on admission to the perinatal center [10, 11]. Since a linear relationship between hemorrhage and increasing SI has been recorded [10], in this study we defined inadequate fluid replacement before admission to the perinatal center as less than SI × 1000 mL of crystalloids. As an indicator of prompt transfer to the perinatal center, we investigated the duration from delivery to arrival at the perinatal center. The duration was calculated roughly by increments of 10 minutes from delivery to arrival at each perinatal center. A prompt transfusion therapy was defined as simultaneous blood transfusion on admission to the perinatal center. In cases of a delay in transfusion, the approximate time taken from admission to the start of blood transfusion was extracted from the medical records. The time was also roughly calculated by increments of 10 minutes from admission to the start of the blood transfusion. In cases involving an inborn delivery, SI on diagnosis was used. A prompt transfusion therapy was defined as rapid blood transfusion soon after diagnosis of uterine inversion (less than 10 minutes). In cases of a delay, the approximate time taken from diagnosis to the start of blood transfusion was extracted from the medical records.

Data are expressed as number (range) or mean ± SD (range).

## 3. Results

According to the medical records investigated, all six women were primiparous and the average maternal age was 28.0 years (Table 1). The average gestational age at delivery was 38.8 ± 1.6 weeks (37~41 weeks); three of the six cases involved vacuum extraction, and two of the six cases included attempted manual removal of the placenta. Except for cases involved attempting manual removal of placenta, umbilical cord traction was done without an elevation of uterus by the hand. The average birth weight at delivery was 3063 ± 191 g (2998~3410 g). In this study, five of the six cases involved an outborn delivery. Only one clinic was located in a rural area and thereby resulted in a geographical disadvantage for transfer (more than 40 minutes by ambulance). The other clinics were all located near a perinatal center (within 10 minutes by ambulance).

Table 2 shows a comparison of the risk profile among the study group. Two cases involved manual removal of the placenta followed by immediate notice of a mass in the vagina or inverted uterus. At the time of notice, they had severe abdominal pain; however, they did not exhibit shock sign such as loss of conscious or abrupt decline in blood pressure. The other four cases were overlooked at first followed by increasing vaginal bleeding. Five of the six cases showed a delay in transfer (more than 50 minutes after delivery, range: 20–100 minutes) and received inadequate fluid replacement. The SI of all cases was at least 1.6 (range: 1.6–2.7) on admission. Five of the six cases involved simultaneous blood transfusion on admission, and the remaining case showed a delay (30 minutes from admission to start of transfusion). All six cases involved successful uterine replacement soon after admission to the respective perinatal center. Two cases (Cases 1 and 5) received manual replacement without uterine relaxing agent at delivery room; the others received manual replacement in operative room with uterine relaxing agent either inhaled sevoflurane or low-dose nitroglycerin (0.1 mg increment dose). The average estimated blood loss was 4578 ± 2143 mL (2750~8367 mL). Four of the six cases included ICU care. Finally, one maternal death occurred due to inappropriate practices that included delay in diagnosis, delay in transfer, inadequate fluid replacement, and delayed transfusion (Case 1). Two patients were subjected to inappropriate practices involving delay in diagnosis, delay in transfer, and inadequate fluid replacement survived (Cases 2 and 5).

## 4. Discussion

In our region, 80% of deliveries occur at a private clinic [9] and blood products are not located on-site at each clinic. As a consequence, private clinics refer a patient to a high-order perinatal center in the event of postpartum hemorrhage such as uterine inversion. This is the setting of the six cases of uterine inversion investigated in this study. Our six cases included one maternal death that resulted from a delay in diagnosis, delay in transfer, inadequate fluid replacement, and failure of a prompt blood transfusion. On the other hand, two patients were subjected to inappropriate practices involving delay in diagnosis, delay in transfer, and inadequate fluid replacement survived. The difference between survivors and death was the presence of a prompt blood transfusion. In a life-threatening situation involving delay in diagnosis, delay in transfer, and inadequate fluid replacement, the administration of a prompt blood transfusion on-site may be critical. Therefore, problems in our region that affect mortality and morbidity of uterine inversion have emerged from this study.

Despite widespread familiarity with uterine inversion, this condition still occurs at a certain frequency and remains one of the life-threatening causes of obstetrical hemorrhage [5]. Diagnosis of uterine inversion is based on clinical signs such as severe abdominal pain, shock with or without profound bleeding, absence of uterine fundus, or presence of a mass in the vagina by pelvic examination. These clinical signs allow us to identify and undergo early replacement of uterus in most cases, followed by causing in uneventful course [4], but there were still four cases of delayed diagnosis in this study. Delay in diagnosis produced a series of negative responses involving delay in replacement of inverted uterus, transfer, and lack of adequate fluid replacement until admission to the perinatal center. Our study revealed that these factors resulted in a critical condition involving either high SI (2.7) or death. Thus, the primary factor that influences mortality and morbidity is still the early recognition of an inverted uterus [12, 13]. However, it is well known that neurogenic shock as one of the early signs of uterine inversion contributes increasing the number of mortality and morbidity [14]. In our study, there were no cases of neurogenic shock at the time of notice. Accordingly, we could not define the importance of neurogenic shock for major factors to define the prognosis in this study.

In general, practitioners had little experience with this condition due to its rarity and therefore did not recognize signs of uterine inversion [15]. Furthermore, most private clinics in Japan have inadequate obstetric services in terms of medical staff [16]. The presence of fewer obstetricians in clinics might therefore reduce the possibility of early recognition. In particular, because of those, it is crucial to recognize the importance of appropriate management of third stage of labor such as avoiding umbilical cord traction, prompt diagnosis, and prompt replacement of inverted uterus. Those practices will yield an uneventful course. It is therefore necessary to create disease-specific guidelines for rare events in obstetrical hemorrhage such as uterine inversion or amniotic fluid embolism.

In cases of a life-threatening situation involving a delay in diagnosis, delay in transfer, and inadequate fluid replacement, the administration of a prompt blood transfusion on-site may be critical. It has also been proposed that a delay in blood transfusion results in coagulopathy and organ failure [17]. Bonnet and colleagues reported that a delay in blood transfusion was associated with a series of maternal deaths from postpartum hemorrhage [18]. They indicated that there is a room for improvement concerning blood transfusion in terms of timing and reported that 79% of the maternal deaths occurred in clinics with an on-site blood bank. In our study, every perinatal center had access to blood products. One facility started blood transfusion with blood-type compatible products following a delay in transfusion (30 minutes from admission to the start of transfusion). The others started blood transfusion with O-group blood that may be transfused into people of all (blood) groups. According to the guidelines of the Royal College of Obstetricians and Gynecologists, O Rh D negative red cells can be given to patients in extreme situations [19]. Therefore, it is assumed that the decision regarding timing and transfusion using O-group blood is the important factor that determines the outcomes. However, our study involved a small number of cases. There is therefore a need to expand the survey of mothers suffering from uterine inversion.

In conclusion, we demonstrated the importance of the monitoring of critical care for uterine inversion in obstetrical hemorrhage. In case of a delayed diagnosis, the findings of our study will result in better obstetrical practices. A delay in diagnosis of inverted uterus results in a life-threatening situation involving a delay in early replacement, transfer, and inadequate fluid replacement. In the case of a life-threatening situation, a prompt blood transfusion using O-group blood on-site is a critical factor that determines the outcome of uterine inversion.

## Figures and Tables

**Table 1:** Demographic data of women with uterine inversion. Results are expressed as the number of individuals. GA: gestational age. ^*∗*^Geographical disadvantage for transfer (more than 40 minutes by ambulance car).

Case	Age (ys)	Parity/abortion	Inborn/outborn	GA at delivery (weeks)	Vacuum extraction	Birth weight (g)	Manual removal of placenta
1	33	0/0	Outborn	37	Yes	2836	No
2	28	0/0	Outborn	39	No	3410	No
3^*∗*^	24	0/1	Outborn	40	Yes	2998	Yes
4	29	0/0	Outborn	37	No	3022	Yes
5	27	0/1	Outborn	41	Yes	3104	No
6	27	0/0	Inborn	39	No	3005	No

**Table 2:** Comparison of the resuscitate profile and outcome among the group with uterine inversion. Results are expressed as the number of individuals. ^*∗*^Geographical disadvantage for transfer (more than 40 minutes by ambulance car). SI: shock index, min.: minutes. Inadequate fluid replacement before admission at the perinatal center was defined as less than SI × 1000 mL of crystalloids.

Case	Delay in diagnosis	Delay in transfer	Inadequate fluid replacement	SI on admission	Delay in blood transfusion	Blood loss (ml)	ICU care	Dead/alive
1	Yes	50 min.	Yes	1.8	30 min.	8367	Yes	Dead
2	Yes	70 min.	Yes	1.6	No	2750	No	Alive
3^*∗*^	No	70 min.	No	2.1	No	5831	Yes	Alive
4	No	80 min.	No	1.7	No	3800	No	Alive
5	Yes	100 min.	Yes	2.7	No	3121	Yes	Alive
6	Yes	20 min.	Yes	2.1	No	3598	Yes	Alive
